# Working hours, side work, and depressive symptoms in physicians: A nationwide cross‐sectional study in Japan

**DOI:** 10.1002/1348-9585.12377

**Published:** 2022-12-02

**Authors:** Makoto Okawara, Tomohiro Ishimaru, Toru Yoshikawa, Michiko Kido, Yoshifumi Nakashima, Anna Nakayasu, Kokuto Kimori, Satoshi Imamura, Kichiro Matsumoto

**Affiliations:** ^1^ Department of Environmental Epidemiology, Institute of Industrial Ecological Sciences University of Occupational and Environmental Health, Japan Kitakyushu Japan; ^2^ Research Center for the Overwork‐Related Disorders National Institute of Occupational Safety and Health, Japan Kawasaki Japan; ^3^ Department of Obstetrics & Gynecology Japanese Red Cross Medical Center Tokyo Japan; ^4^ Department of Psychiatry Mitsui Memorial Hospital Tokyo Japan; ^5^ Executive Boards Japan Medical Association Tokyo Japan; ^6^ Imamura Clinic Tokyo Japan; ^7^ Japan Medical Association Tokyo Japan

**Keywords:** health care services, mental health, occupational health, physicians

## Abstract

**Objectives:**

Long working hours in Japan is a social concern. This is also true for the working hours of physicians, who often do side work in addition to working at their main hospital. New physician work regulations to be implemented in Japan require work‐hour management, including side work. This study examined the association between physicians' overtime and side work hours and their health outcomes.

**Methods:**

We conducted a cross‐sectional study using data from a survey of working physicians conducted by the Japan Medical Association from 2021 to 2022. Questionnaires completed by 2906 full‐time working physicians aged 24–69, excluding junior resident doctors, were analyzed. Depressive symptoms and the presence of thoughts of death or suicide using the Quick Inventory of Depressive Symptomatology ‐ Japanese version was used to assess health outcomes. Logistic regression analysis was performed using overtime at the main place of employment and side work hours as exposure factors.

**Results:**

Both depressive symptoms and thoughts of death or suicide increased in the group with longer overtime hours at the main place of work. A similar tendency was observed for side work but adjusting for overtime hours eliminated the association. In other words, total working hours had an impact on health, regardless of whether overtime work at the main place of employment or side work.

**Conclusions:**

Despite the existence of multiple hurdles to managing working hours, including side work, the working environment for physicians needs to be improved to protect their health and quality of medical care.

## 1 INTRODUCTION

It is important to ensure the health of physicians, both for their own well‐being and for the quality of health care services. It is estimated that internationally 28% of physicians have burnout and 24% have depressive symptoms.^1^ One in 16 surgeons in the U.S. have thought about suicide, and suicidal ideation among physicians is higher than in the general population.^2^, ^3^, ^4^ The risk of suicide is particularly high for female physicians, and it has been noted that physician suicides often occur later in life than in the general population.^5^ Suicide among physicians is associated with depression and burnout.^6^, ^7^ Although many studies have shown that the risk of physician burnout is higher in women, it is higher at younger ages, in contrast to the risk of suicide with respect to age.^8^ Physicians' hard work and career plans have been noted to conflict with their families interests, and the harshness and dissatisfaction of physicians' work affects their mental health.^9^, ^10^, ^11^, ^12^ When a physician's health is poor, the ability to work function adequately may be adversely affected, with repercussions for the quality of health care services. For example, a physician's depressive symptoms may impair his or her ability to think and make decisions. The impact of burnout on individual physician health and treatment outcomes is considerable.^12^, ^13^ It also has economic consequences.^14^ Preventing health problems for physicians will help them maintain their work function and benefit the health and safety of their patients.

Physicians' long working hours are an important and necessary issue to be solved for the health of physicians. A 2019 survey in Japan revealed that about 40% of physicians worked more than 60 h/week and 10% worked more than 80 h/week.^15^ Overwork‐related disorders are a public health issue,^16^, ^17^ and can lead to deaths, including suicides, due to depressive symptoms or burnout.^18^ Long working hours increase the risk of developing depressive symptoms.^19^, ^20^ Physician burnout is associated with long working hours and is gaining recognition as a global crisis.^8^, ^21^ Furthermore, these conditions may be exacerbated during the COVID‐19 pandemic due to worsening working conditions.^22^, ^23^ It is important to monitor physicians' working hours and take appropriate actions to avoid health problems.

In Japan, preparations are underway to manage physicians' excess working hours, focusing on multiple works, mainly side work. Many Japanese physicians do part‐time side work at hospitals other than the main hospital at which they are employed, providing care at night and on weekends. Indeed, a large survey revealed that 60% of full‐time hospital physicians and 90% of physicians at university hospitals do side work.^24^ Until now, management of physicians' working hours has been limited to their main place of employment; side work has not been considered. In 2024, after a period of transition for the law to take effect, physicians' main hospitals will be required to check and keep track of their total hours worked. However, the association between physicians' work hours, including side work, and their health is largely unknown.

We hypothesized that overtime at physicians' main hospital and side work hours affect physicians' health. The purpose of this study was to determine whether physicians' total working hours, including overtime and side work, were associated with depressive symptoms and thoughts about death or suicide.

## 2 MATERIALS AND METHODS

This cross‐sectional study drew on data from the Third Survey on the Current State of Employed Physicians' Health and Support, conducted by the Japan Medical Association (JMA) between December 9, 2021, and January 31, 2022. With approximately 170 000 members, the JMA is the largest professional organization of physicians in Japan. Member surveys are conducted regularly, details of which are presented elsewhere.^25^ Participants responded anonymously, by post or online. One reminder postcard was sent before the survey closed. This study was approved by the Research Ethics Committee of the National Institute of Occupational Safety and Health, Japan (2021N32).

We sampled 21 737 employed physicians, all of whom were members of the JMA. Sampling was conducted in two phases. First, we used a random sample of 10 000 among 61 122 physicians. Then, we sampled 11 737 of 14 029 employed physicians below 40 years of age, excluding 2292 already sampled in the first phase. The reason for the additional sampling of physicians younger than 40 was that previous surveys led us to expect a lower response rate from younger physicians. In general, the retirement age for Japanese is 60 or 65, but in some cases, physicians continue to work at the same hospital even after reaching retirement age, mainly as hospital administrators. As age increases, however, the content and pace of work are adjusted seamlessly. We excluded junior residents and physicians over 70 years of age, as these groups' work styles are considered different from that of regular physicians; our sample thus included full‐time physicians aged between 24 and 69 years old, regardless of whether they worked at private or public hospitals.

### 2.1 Assessment of depressive symptoms and thoughts of death or suicide

Depressive symptoms were measured using the Quick Inventory of Depressive Symptomatology Japanese version (QIDS‐J).^26^, ^27^ Based on previous studies, respondents with a total QIDS score of 16 to 20 were defined as having severe depressive symptoms, and a score of 21 to 27 as having very severe depressive symptoms. For this study, we defined those with severe or very severe depressive symptom scores as “having depressive symptoms”. Thoughts of death or suicide were measured using one of the QIDS questions, item #12: (0) “Does not think of suicide or death,” (1) “Feels life is empty or is not worth living,” (2) “Thinks of suicide/death several times a week for several minutes,” (3) “Thinks of suicide/death several times a day in depth, or has made specific plans, or attempted suicide”. Of the four options, a response of 2 or 3 was defined as having thoughts of death or suicide. Although several studies have used item #12 in QIDS‐SR as “suicidal ideation,”^28^, ^29^, ^30^, ^31^ there are concerns that the assessment of suicidal ideation using this single item may result in overestimation compared to clinician‐ascertained suicidal ideation.^32^ In addition, given that the participants in this study were physicians working during the COVID‐19 pandemic, with more than usual exposure to death, the use of only this item might not have been sufficiently validated. Therefore, we called this outcome “thoughts of death or suicide” rather than “suicidal ideation.”

### 2.2 Assessment of working hours and other covariates

Main overtime and side‐work hours were measured by categorical responses to the following questions. For overtime work hours at main hospital: “During the past month, please indicate the number of hours of overtime work at your main hospital. (Overtime, work on holidays, etc. regardless of whether you reported or not. However, excluding hours spent on overnight duty or day duty that can be excluded from working hours).” For side‐work hours: “During the past month, please indicate the number of hours you engaged in side work outside of your primary employer's working hours (e.g., working nights or holidays outside of your main hospital.)” Eight response options were available: none, less than 20, 20–39, 40–59, 60–79, 80–99, 100–119, and 120 h or more. Responses were categorized into three categories: 0–39 h (few), 40–79 h (moderate), and 80 h or more (many).

The following items were included as covariates: sex, age, specialty, number of working days per week (<3.5, 4–4.5, 5, and >5.5), and number of annual paid leave days the respondent really had. Specialty was categorized into (1) internal medicine, (2) surgery, (3) pediatrics/gynecology, (4) psychiatry/psychosomatic medicine, and (5) other.

### 2.3 Statistical analysis

Data with missing values for any variables were excluded from the analysis. A multivariate logistic regression analysis was performed using the presence or absence of severe depression and thoughts of death or suicide as outcomes and overtime work hours at main hospital or side work hours as exposure factors. In model 1, covariates for multivariate analysis were sex, age, specialty, working days per week, and accurate number of annual paid leave days. In model 2, to examine the effect of total working hours considering both overtime and side work hours, both of these hours were added to model 1. Stata 16.1 (Stata Corp) was used for the analysis. Any effect at *P* < .05 was considered significant.

## 3 RESULTS

A total of 4032 (17.7%) respondents participated in the study, and the data from 2906 who met the inclusion criteria were analyzed; however, 83 (2.9%) of the latter group were excluded due to one or more missing QIDS scores or other variables used in the analysis (Table S1), ultimately leaving 2823 individuals whose data were included in the final analysis. Table 1 shows the demographics of the participants. Their ages were relatively evenly distributed. About 50% of the sample reported taking less than the minimum Japanese legal requirement of 5 days of annual paid leave in a year. Defining long hours as more than 80 h/month revealed that 10.5% of physicians worked long overtime hours at their main hospitals, while 4.4% worked long hours of side work.

**TABLE 1:** Participants' demographic and sociological characteristics.

	*N* = 2823	
Variables	***n***	%
Age, years, median (interquartile range)	51 (37–60)	
20–29	175	6.2%
30–39	739	26.2%
40–49	416	14.7%
50–59	741	26.2%
60–69	752	26.6%
Sex, men	2135	75.6%
Specialty		
Internal medicine	1090	38.6%
Surgery	596	21.1%
Pediatrics/gynecology	385	13.6%
Psychiatry/psychosomatic medicine	203	7.2%
Other	549	19.4%
Number of working days per week^a^		
≤3.5	180	6.4%
4–4.5	560	19.8%
5	1582	56.0%
≥5.5	501	17.7%
Number of annual paid leave days actually obtained (days per year)		
0	534	18.9%
1–4	866	30.7%
≥5	1423	50.4%
Overtime at main hospital (hours per month)		
0–39	1905	67.5%
40–79	621	22.0%
≥80	297	10.5%
Side work hours (hours per month)		
0–39	2450	86.8%
40–79	248	8.8%
≥80	125	4.4%
Severe or very severe depressive symptoms (QIDS score ≥ 16)	72	2.6%
Thoughts of death or suicide (QIDS‐J, item #12)	118	4.2%
Thinks of suicide/death several times a week for several minutes	102	3.6%
Thinks of suicide/death several times a day in depth, or has made specific plans, or attempted suicide	16	0.6%

^a^ The basic working schedule in Japan is 8 hours a day, 40 hours a week.

Table 2 shows the association between overtime work hours at their main hospitals or side‐work hours and physicians' severe depressive symptoms or thoughts of death or suicide. In model 2, the group with many hours of main hospital overtime (80 h or more) had a significantly higher rate of severe depression (adjusted odds ratio [aOR]: 4.24, 95% confidence interval [CI]:2.22–8.09, *P* < .001) and thoughts of death or suicide (aOR: 2.40, 95% CI: 1.42–4.06, *P* = .001) than the group with 0–39 h of overtime. The trend test indicated a similar outcome, and these were similar for models 1 and 2. Concerning side work, the moderate (40–79 h) side‐work group reported significantly higher rates of severe depressive symptoms and thoughts of death or suicide in model 1 compared to the 0–39 h group. By contrast, model 2, which included overtime hours as a covariate, revealed no significant association between side‐work hours and health outcomes.

**TABLE 2:** Association between physicians' working hours (overtime at main hospital and side work) and health outcomes.

			*N* = 2823	Outcome observed	Univariate				Model 1^a^				Model 2^b^			
Outcome variables	Explanatory variables		%	%	OR	95% CI		*P*	OR	95% CI		*P*	OR	95% CI		*P*
Severe or very severe depressive symptoms																
(QIDS ≥ 16)	Overtime at main hospital	0–39	67.5%	1.3%	Reference			<.001	Reference			<.001	Reference			<.001^c^
		40–79	22.0%	4.0%	3.29	1.86	5.80	<.001	2.92	1.62	5.28	<.001	2.79	1.55	5.05	.001
		≥80	10.5%	7.7%	6.58	3.66	11.81	<.001	4.67	2.47	8.82	<.001	4.24	2.22	8.09	<.001
	Side work hours	0–39	86.8%	2.2%	Reference			.005	Reference			.013	Reference			.084^c^
		40–79	8.8%	4.8%	2.26	1.19	4.28	.013	2.33	1.18	4.58	.015	1.78	0.89	3.54	.102
		≥80	4.4%	4.8%	2.24	0.94	5.30	.068	2.08	0.83	5.23	.118	1.73	0.68	4.42	.252
Thoughts of death or suicide	Overtime at main hospital	0–39	67.5%	3.2%	Reference			<.001	Reference			.001	Reference			.001^c^
		40–79	22.0%	5.2%	1.64	1.06	2.54	.026	1.63	1.04	2.56	.035	1.57	1.00	2.48	.050
		≥80	10.5%	8.4%	2.77	1.71	4.50	<.001	2.56	1.53	4.31	<.001	2.40	1.42	4.06	.001
	Side work hours	0–39	86.8%	3.8%	Reference			.026	Reference			.030	Reference			.090^c^
		40–79	8.8%	6.5%	1.73	1.00	2.99	.050	1.80	1.02	3.20	.044	1.56	0.87	2.78	.134
		≥80	4.4%	6.4%	1.71	0.81	3.61	.157	1.76	0.80	3.87	.158	1.59	0.72	3.51	.251

^a^ Adjusted for sex, age, specialty, working days per week, and number of annual paid leave days actually obtained (days per year).

^b^ In addition to model 1, the model includes overtime at main hospital and side work hours as covariates.

^c^ *P* for trend.

## 4 DISCUSSION

This study investigated the association between physicians' working hours and health outcomes. The results showed that depressive symptoms and thoughts of death or suicide tended to increase with longer working hours, with the association between overtime work at the main employing hospital and health outcomes being particularly robust.

The strong association between long working hours at the physician's main hospital and severe depressive symptoms and thoughts of death or suicide is in agreement with several previous studies.^33^, ^34^ Compromised sleep duration and work‐life balance are involved in the mechanism by which long work hours lead to depression.^35^ Longer working hours make it harder to get enough sleep,^36^ and the circadian rhythm is disrupted by a shifting of the timing of sleep.^37^ Furthermore, more working hours mean less free time, which creates an unsatisfactory work‐life balance.^38^ These psychological and physical stresses may underlie the reported depressive symptoms and thoughts of death or suicide.

The results indicated a significant association between moderate side work and depressive symptoms and thoughts of death or suicide; however, significance disappeared when overtime hours at the main hospital were added as a covariate. In other words, side work itself is not a unique risk factor that negatively affects health; instead, it is longer total work hours, regardless of whether through side work or overtime work at the main hospital, that can negatively affect health. Overtime hours at the main hospital were a strong contributor to negative health outcomes. In fact, the percentage of physicians working long hours at the main hospital was more than double that of those involved in long side‐work hours, and prevalence of negative outcomes was greater in the former group.

Management of maximum working hours for physicians is due to begin in 2024, including side work. Nevertheless, there are a number of hurdles to overcome in this move. Japanese physicians engage in two types of side work. One type consists of individual, voluntarily work unrelated to their main hospital, to maintain and improve one's abilities, or for money. The other type is when a physician employed at a university or regional core hospital does side work at an affiliated hospital, mediated by their main hospital, to ensure consistent supply of medical care at the smaller, usually regional hospital, or to get the chance to perform surgery or other techniques. This latter type of side work is often done by young physicians after their junior resident program. It also allows young physicians working in university hospitals who are paid less than if they worked in general hospitals to supplement their income. Hurdles to managing maximum working hours, including side work, exist mainly for young physicians, and also for the institutions involved that are mediated by the main hospitals of these physicians. If maximum working hours, including side work, are capped while overtime at main hospitals remains heavy, side work will have to be restricted. Likely disadvantages of such a move include the inability to maintain local medical care systems, restricted opportunities for young physicians to improve their ability, and reduced income for physicians. Regulation of resident doctors' working hours has been addressed in several countries with the aim of protecting their health and ensuring safety in medical services. Nevertheless, concerns remain that these regulations may lead to a lack of training for resident doctors and a lack of medical service provision. No uniform practice has been established, and countries are handling the issue with reference to their own particular circumstances.^39^

There are three ways to remove the obstacles to capping working hours, including side work. First, overtime at the main hospital can be reduced by updating work practices. If the system in which a single physician is in charge of a patient 24 h a day can be changed, and if rules for physicians' working styles can be established and updated by promoting a team‐charge system and a shift system to reduce overtime, physicians will be able to pursue important side work. Second, focus can be placed on reducing the burden associated with side work. In Japan, a hospital may apply to the chief of the Labor Standards Bureau for exemption from working hours on overnight or day duties for which physicians are rarely called. Cooperation between main hospitals and hospitals requiring side workers to assign less burdensome overnight duty side work to physicians who already have a heavy overtime load at their main hospital, and applying for work hour exemptions, may solve the problem of physician income. Third, the nation can come to a consensus in recognizing that medical resources are finite. In Japan, patients often seek medical care at night or on holidays for non‐urgent conditions, or request emergency medical services.^40^ A telephone consultation service for pediatric patients, a telephone service (#7119) and a smartphone application to assist in determining the need for emergency calls have been developed, but as of October 2020, the population coverage rate of #7119 is 46%, which is not sufficient.^41^ It is important to reduce the burden on medical institutions through dissemination and increasing awareness of these measures, and to achieve this, cooperation among physicians, medical institutions, and the public is required.

Even with the above‐mentioned measures, young physicians in particular may still be involved in long working hours, to improve their skills, especially in areas with fragile medical provision. In the context of such difficult situations, health support programs will be implemented in the 2024 revision of the physician's work system, alongside long‐term plans to reduce working hours. There will be a 28‐h limit to consecutive working and at least a 9‐h interval between work shifts; provisions for compensatory rest; and periodic monitoring of sleep debt and fatigue levels for physicians who work long hours. A recent study found an association between Japanese physicians' sleep duration and high levels of stress and depressive symptoms, indicating the importance of sleep duration rather than working hours.^42^ If it is difficult to suddenly reduce side work, another effective measure is to ensure sufficient sleep by managing individual physicians' working conditions and monitoring their health status.

This study has several limitations. First, the procedure involved voluntary participation in a questionnaire administered by members of a specific professional organization, which has sampling‐ and self‐selection bias. The prevalence of depression and thoughts of death or suicide reported here cannot be extended to all Japanese physicians. Second, the working hours were self‐reported, and precise details were unclear, such as whether the job was a high‐workload job or mainly a standby position. Third, the background of the side work and whether it was voluntary or mediated by the main hospital is unknown. Fourth, there are unmeasured potential confounders such as family constitution or perception of economic compensation for work activity. It is unclear how these might affect the results.

Finally, the study were conducted during the COVID‐19 pandemic, and the resulting increased burden on health care workers may have affected working hours, and physicians' mental health and thoughts of death or suicide. For example, it is possible that thoughts about patient deaths, particularly pandemic deaths, was confounded with thoughts of their own death or suicide. Also, given that the QIDS was originally developed to observe the severity of depression in depressed patients, and not to assess working physicians' mental health, the use of QIDS single item #12 was not considered adequately validated. The result should therefore be interpreted with caution.

## 5 CONCLUSION

The long working hours of Japanese physicians were found to be associated with severe depressive symptoms and thoughts of death or suicide. Overtime at the main hospital was especially strongly implicated, but it is also reasonable to consider side work in managing total working hours. The working conditions for physicians in Japan are harsh, and improving them is an important issue for maintaining both physicians' well‐being and the medical service itself. Physicians, medical institutions, and the public need to cooperate to achieve this goal, in order to meet the 2024 cap on working hours that takes into account side‐work hours.

## AUTHOR CONTRIBUTIONS

Toru Yoshikawa planned the overall survey; all authors were involved in the development of the survey methodology and questionnaire; Makoto Okawara conceived the research questions, performed the statistical analysis, and described the first draft of this article; all authors revised and approved final manuscript.

## DISCLOSURE

*Approval of the research protocol*: This study was approved by the Research Ethics Committee of the National Institute of Occupational Safety and Health, Japan (2021 N32). *Informed Consent*: Participation was voluntary and anonymous; therefore, the requirement for written informed consent was waived. *Registry and the Registration No. of the study/trial*: N/A. *Animal Studies*: N/A. *Conflict of Interest*: N/A.

## Supporting information


Table S1.

## Data Availability

Research data are not shared.
