# Supplementary material for: Working hours, side work, and depressive symptoms in physicians: A nationwide cross‐sectional study in Japan
Source: J Occup Health. 2022 Dec 2;64(1):e12377. doi: 10.1002/1348-9585.12377 (PMC9717704; doi:10.1002/1348-9585.12377)
Supplement: Supplementary file 1 — Table S1. [file JOH2-64-e12377-s001.docx]

| Supplementary table. Distribution of missing data | | | |
| --- | --- | --- | --- |
|  |  | N = 2,906 | |
|  |  | Missing data | |
| Variables | | n | % |
| Any of following variables missing | | 83 | 2.9% |
|  |  |  |  |
| Sex | | 10 | 0.3% |
| Age | | 5 | 0.2% |
| Specialty | | 4 | 0.1% |
| Number of working days per week | | 10 | 0.3% |
| Number of annual paid leave days actually obtained | | 19 | 0.7% |
| Overtime at main hospital | | 10 | 0.3% |
| Side work hours | | 6 | 0.2% |
| QIDS score | | 22 | 0.8% |
